# HIV-1 Structural Proteins Serve as PAMPs for TLR2 Heterodimers Significantly Increasing Infection and Innate Immune Activation

**DOI:** 10.3389/fimmu.2015.00426

**Published:** 2015-08-19

**Authors:** Bethany M. Henrick, Xiao-Dan Yao, Kenneth Lee Rosenthal, the INFANT study team

**Affiliations:** ^1^Department of Pathology and Molecular Medicine, McMaster Immunology Research Centre, Michael G. DeGroote Institute for Infectious Disease Research, McMaster University, Hamilton, ON, Canada

**Keywords:** human immunodeficiency virus type 1, immune activation, inflammation, innate immunity, pathogen-associated molecular patterns, pattern recognition receptors, toll-like receptors, TLR2 heterodimers

## Abstract

Immune activation is critical to HIV infection and pathogenesis; however, our understanding of HIV innate immune activation remains incomplete. Recently we demonstrated that soluble TLR2 (sTLR2) physically inhibited HIV-induced NFκB activation and inflammation, as well as HIV-1 infection. In light of these findings, we hypothesized that HIV-1 structural proteins may serve as pathogen-associated molecular patterns (PAMPs) for cellular TLR2 heterodimers. These studies made use of primary human T cells and TZMbl cells stably transformed to express TLR2 (TZMbl-2). Our results demonstrated that cells expressing TLR2 showed significantly increased proviral DNA compared to cells lacking TLR2, and mechanistically this may be due to a TLR2-mediated increased CCR5 expression. Importantly, we show that HIV-1 structural proteins, p17, p24, and gp41, act as viral PAMPs signaling through TLR2 and its heterodimers leading to significantly increased immune activation via the NFκB signaling pathway. Using co-immunoprecipitation and a dot blot method, we demonstrated direct protein interactions between these viral PAMPs and TLR2, while only p17 and gp41 bound to TLR1. Specifically, TLR2/1 heterodimer recognized p17 and gp41, while p24 lead to immune activation through TLR2/6. These results were confirmed using TLR2/1 siRNA knock down assays which ablated p17 and gp41-induced cellular activation and through studies of HEK293 cells expressing selected TLRs. Interestingly, our results show in the absence of TLR6, p24 bound to TLR2 and blocked p17 and gp41-induced activation, thus providing a novel mechanism by which HIV-1 can manipulate innate sensing. Taken together, our results identified, for the first time, novel HIV-1 PAMPs that play a role in TLR2-mediated cellular activation and increased proviral DNA. These findings have important implications for our fundamental understanding of HIV-1 immune activation and pathogenesis, as well as HIV-1 vaccine development.

## Introduction

Chronic immune activation and inflammation serve as fundamental drivers of HIV-1 infection, replication, and pathogenesis (1–6). This is supported by the classical observation that pathogenic SIV is associated with high levels of immune activation and pathogenesis leading to death in non-natural non-human primate (NHP) hosts, whereas non-pathogenic SIV infection in natural NHP hosts is associated with low levels of immune activation despite high viral loads (7).

Importantly, chronic immune activation and inflammation, the hallmark of HIV infection, eventually leads to premature co-morbidities, including cardiovascular, neurological, liver, brain, and bone pathologies (8–11). This despite the use of combination antiretroviral therapy (cART), which dramatically reduces systemic viral load, and, we have previously shown, normalizes PBMC TLR expression (12), suggesting that HIV or components thereof are involved in increased and dysfunctional innate immune activation (12).

To this point, HIV-1 structural proteins and glycoproteins have been detected persistently in germinal centers of lymphoid tissue from HIV-infected individuals, including those receiving cART with undetectable viral loads (13–15). Moreover, *in vitro* studies have observed a broad spectrum of effects including, increased proliferation, maturation, cytokine production, cell surface marker expression and HIV-1 replication in PBMC, epithelial, and endothelial cells after exposure to HIV-1 ENV, gp120/160 or matrix protein, p17 (16–20). gp120 plays a role in the immunostimulatory effects related to HIV-1-associated dementia (21). gp41 has been shown to significantly enhance HIV-1 infection and replication *in vitro* (22). Likewise p24 and p17 have been shown to possess potent immunostimulatory properties leading to increased HIV-1 replication in PBMCs of infected individuals receiving cART (23), as well as activated PBMCs *in vitro* (16). p17 has been shown to hijack CXCR2 and syndecan2 by activation of Jak/STAT pathway that is responsible for local activation and recruitment of inflammatory cells in HIV/HCV co-infected or HIV-1 mono-infected patients (24). Furthermore, HIV-1 has been shown to modulate the innate immune system by activating specific pattern recognition receptors (PRRs) in order to enhance viral replication in plasmacytoid dendritic cells (25–27). Despite these fundamental findings, to date, the only well recognized HIV-1 PAMP is uridine-rich HIV-1 ssRNA, which is detected by intracellular TLR7/8 (28).

The innate immune system is the first line of defense against infection and consists of innate immune cells that are able to respond to infectious pathogens by PRRs, defined as innate immune activation (29). Toll-like receptors (TLRs), a family of innate PRRs, play a critical role in the early recognition of pathogens and are largely responsible for activating innate immunity and shaping subsequent adaptive immune responses (30, 31). Typically, the recognition of PAMPs via TLR engagement triggers a signaling cascade resulting in the activation of transcription factor, nuclear factor kappa B (NFκB), leading to the downstream production of anti-viral and pro-inflammatory cytokines (32). NFκB is particularly important during HIV-1 infection since its activation facilitates viral replication by binding the long terminal repeat (LTR) (33).

Therefore, identifying novel interactions between TLRs and HIV will have important implications for our fundamental understanding of HIV-mediated innate immune activation and pathogenesis.

While classically considered in the context of bacterial recognition, TLR2 is unique among the TLR family in that it can heterodimerize with co-receptors TLR1, TLR6, and TLR10 (34, 35), thus profoundly increasing the diversity of PAMPs recognized. Of particular interest, a number of viral proteins have been identified as novel PAMPs for TLR2 including cytomegalovirus (CMV) glycoprotein B (36), herpes simplex virus (HSV) gH/gL and gB (37), hepatitis C virus (HCV) core protein (38), and measles virus hemagglutinin A glycoprotein (39). Specifically regarding HIV, Thibault et al. (40) described that detection of pathogen-derived products through TLR2 induced an effector phenotype in naïve and memory CD4+ T cells and increased HIV replication (40). Conversely, the extracellular portion of TLR2, which is found systemically and in mucosal fluids, significantly inhibits pro-inflammatory cytokine production (41–43) and directly inhibits cell-free HIV-1 infection *in vitro* (44, 45). Moreover, we previously demonstrated that soluble TLR2 (sTLR2) directly interacts with HIV-1 p17, p24, and gp41 and inhibits viral protein-induced NFκB activation and inflammation (45). These findings led us to further hypothesize that structural proteins of HIV-1 may serve as PAMPs for cellular TLR2 heterodimers.

In this paper, we report significantly increased HIV-1 provirus in TLR2-bearing cells compared to cells that do not express TLR2. Our data also demonstrates that HIV-1 p17, p24, and gp41 directly bind to TLR2 and significantly increase cellular activation *in vitro*, an effect that was knocked down by TLR2-specific siRNA or anti-TLR2 antibodies. Lastly, our data identified an NFκB signaling pathway responsible for the increased pro-inflammatory cytokine production and significantly increased CCR5 expression observed in primary human T cells.

## Materials and Methods

### Cell lines and reagents

TZMbl (JC53-BL) cells (Dr. D. Montefiori, Duke University, North Carolina) and TZMbl-2 were cultured in DMEM without and with 0.8 mg/mL geneticin (G418; Invitrogen, Burlington, ON, Canada), respectively, as described previously. HEK293-TLR2/1 and TLR2/6 (InvivoGen, Burlington, ON, Canada) were cultured as previously described (44). HIV-1 proteins included p17 (Virogen, Mississauga, ON, Canada), p24, gp41, nef (Genway Biotech, Inc., San Diego, CA, USA), and gp120 kindly provided by NIH AIDS Research and Reference Reagent Program. TLR ligands, ssRNA40 (Mobix, McMaster University), LPS, Pam_3_CSK_4_ (InvivoGen), and poly I:C (Sigma-Aldrich, Burlington, ON, Canada) were diluted in phosphate-buffered saline (PBS).

### Establishment of a stable TLR2 transfected TZMbl-2 cell line

Human TLR2 cDNA was generated by RT-PCR from PBMC and cloned into pcDNA3.1(+)-002 (kindly provided by Dr. Jonathan Bramson, McMaster University). The construct was used as a template for PCR-amplification of the TLR2 gene and inserted into pIRES2-ZsGreen1 vector (Clontech, Burlington, ON, Canada), named pIhT2G. DNA sequencing confirmed its structure, and TLR2 protein expression was detected using western blot analysis. The plasmid was transfected into TZMbl cells with Lipofectamine 2000 (Invitrogen, Burlington, ON, Canada) according to the manufacturer’s instructions. Three rounds of selection for single transfected cells were completed with medium containing 0.8 mg/mL G418. Long-term expression of TLR2 was confirmed using RT-PCR and western blot analyses up to passage 15. All experiments were completed in cells before this mark. Importantly, the confirmatory use of TZMbl-2 was a rationale choice given its stable expression of canonical HIV-1 receptors and Tat-regulated reporter genes, which allowed for sensitive and reproducible quantification of HIV-1 integration (46).

### Viral stocks and HIV-1 integration assay

HIV-1 R5-tropic BaL was prepared and tissue culture infectious doses (TCIDs) of pooled supernatants as well as *in vitro* functional assays were determined using TZMbl and TZMbl-2 cells, as previously described (47). Optimal time for cell-free R5 HIV-1 proviral DNA was determined using qRT-PCR analysis of the Pol gene prior to infection studies and found to be 8 h post exposure in TZMbl cells (Figure S2 in Supplementary Material).

TZMbl cells were plated in 24-well plates and left overnight. At approximately 80% confluence, cells were transfected for 24 h with Lipofectamine 2000 containing 0.5 μg/well of plasmid phTLR2 or phTLR3, which was generated in the same way as phTLR2, or vector pcDNA3.1(+)-002m, respectively. The cells were superinfected with 100 TCID_50_ HIV-1 for 48 h. Total cellular DNA was extracted for analysis of relative integration by qPCR of HIV-1 pol and18S rRNA genes.

CD4+ T cells were enriched from donated human PBMC with EasySep Human Enrichment CD4+ T cell Kit (Stemcell). The CD4+ T cells were stimulated with 5 μg/mL phytohemagglutinin (PHA) – 20 U/mL IL2 – RPMI20 for 3 days. The cells were then pre-treated with RPMI10 containing 5 μg/mL polybrene (Sigma) and 5 μg/mL antibody cocktails, either a mixture of non-specific antibodies (normal rabbit and goat IgG sc-2027, sc-2028) or a mixture of TLR2 antibodies (IMG-410A, sc-8689, sc-21760x, and R&D AF2616) at 37°C for 1 h, followed by infection with HIV-1 300 TCID50/well in a 96-well plate overnight. 5 μg/mL infuvirtide (T20), HIV-1 inhibitor, was used as a negative control. Media was removed and replaced with RPMI20 containing the same antibody cocktails for a total of 6 days. DNA was extracted with 0.2 mg/mL proteinase K-CLB (1.2%SDS–50 mM Tris-HCl pH8.0–4 mM EDTA–4 mM CaCl_2_) and phenol. Relative proviral DNA was then assessed by qPCR.

### Quantitative reverse-transcriptase real-time polymerase chain reaction and conventional RT-PCR

Total RNA was extracted from TRIzol samples as previously reported (48). Reverse transcription (RT) reactions and qRT-PCR of selected genes and internal controls, RPL13A or 18S rRNA gene, were completed as described previously (48). PCR primers for COX-2, TNF-α, CCR5, and HIV Pol were designed using the program, Primer 3.0 (http://frodo.wi.mit.edu), and supplied by Mobix (McMaster University, Hamilton, ON, Canada). Total RNA was harvested from all cells used in this study and mRNA levels for TLR1, 2, 3, 4, 6, 10 and β-actin were assessed using conventional RT-PCR.

### Immunoassays

Endotoxin detection assay (kindly provided by Dr. Dawn Bowdish, McMaster University) was completed according to the manufacturer’s instructions (Lonza, Rochester, NY, USA). OptiEIA was used to measure IL-8 levels in cell culture supernatants according to manufacturer’s instructions (BD Biosciences).

### Small interfering RNA knockdown

siRNA molecules were used to knockdown human TLR1 (Sigma-Aldrich, SASI_Hs01_00162170/AS), TLR2 (Sigma-Aldrich, SASI_Hs01_ 00081589/AS) or non-targeting siRNA (Invitrogen, 129201 H07/129296 H05) as previously described (49) for 48 h before exposure to specific HIV-1 proteins, and supernatants were collected at 18 h for pro-inflammatory cytokine production.

### Dot blot assay

20 pmol of HIV proteins, test reagents (Cell Sciences), and negative controls (urea and glutathione transferase enzyme tag; Virogen) were blotted onto nitrocellulose membrane as previously described (50), and incubated with the cytosol or cell membrane fractions of either TZMbl or TZMbl-2, probed with anti-TLR1 or anti-TLR2 antibodies, and detected with Femto chemiluminescent substrates (Thermo).

### Co-immunoprecipitation

The plasmid, pIhT2G was subcloned by removing the TLR2 stop code and inserting an oligonucleotide containing three repeated Flag-tags and a stop code, which was PCR-amplified from p3 × Flag-CMV-7.1 (Sigma) as a template, generating TLR2-flag expression plasmid, pIhT2FG. This plasmid was transfected with Lipofectamine 2000 into TZMbl or HEK293 cells for 2 days and TLR2-Flag expression was confirmed by western blot with anti-TLR2 (R&D) and anti-Flag (Sigma) antibodies, respectively. The bulk prepared TLR2-Flag lysates and CD14 lysates, pCD14 kindly provided by Evelyn Kurt-Jones (Univ. Massachusetts Med. School, Worcester, MA, USA), with 20 mM CHAPS, 3-((3-cholamidopropyl)dimethylammoniol)-1-propanesulfonate, in a binding washing buffer (BWB) containing 8 mM NaPO_4_–2 mM KPO_4_–10 mM KCl–140 mM NaCl from HEK293 cells were used as resource of TLR2 protein in co-IP. The mixture of 50 μg cell lysates containing either TLR2-Flag or none with individual 10 μg pure HIV-1 proteins, p17, p24, or gp41, was rotated in BWB at 4°C overnight. The positive control was a reaction of cell lysates containing TLR2-Flag and CD14 in presence of 10 μg/mL Pam_3_CSK_4_. Then anti-Flag, anti-p24 (sc-69728), anti-gp41 (GWB-22A6B9), and anti-CD14 (sc-58951) antibodies were added respectively and rotated at 4°C for an additional 2 h. The complexes were pulled down with 10 μL Dynabeads protein G (Invitrogen). The beads were washed three times with BWB on a 96-well magnet stand. Finally the beads were boiled in BWB and subjected to WB. The membranes were probed with anti-p17 (sc-69725) and anti-Flag antibodies, respectively.

### Western blot

TZMbl-2 nuclei were isolated as previously described (51). Primary T cells were separated from human PBMC with CD3 positive selection Kit (StemCell Technologies). Cell lysate, membrane, cytosol, and nuclear pellet total protein were evaluated using DC assay (Bio-Rad) and run on SDS-PAGE gels. Primary antibodies included: anti-TLR1, anti-TLR2 (R&D Systems) and anti-TLR2, anti-TLR6 (Santa Cruz Biotechnology), anti-β-actin, and anti-Histone 3 (Cell Sciences) antibodies. Secondary antibodies included: HRP-labeled donkey anti-goat IgG, HRP-labeled chicken anti-mouse IgG (Santa Cruz Biotechnology), HRP-labeled mouse anti-rabbit IgG (Pierce Biotechnology Inc.), and goat anti-rabbit IgG-HRP (Bio-Rad).

### Statistical analysis

All statistical analyses were performed using GraphPad Prism 5.0 software (GraphPad). The Mann–Whitney *U*-test was used to directly compare two groups for HIV integration, immune responses, cellular activation, and CCR5 expression. All *P*-values are two-tailed and considered statistically significant if *P* < 0.05.

## Results

### Characterization of TLR expression in TZMbl cells and establishment of a functional stably transformed TLR2 cell line, TZMbl-2

In order to investigate the effect cellular TLR2 expression has on host innate responses to HIV-1 proteins and infection, we initially set out to utilize the well-characterized HIV-1 luciferase reporter assay, TZMbl cell line (46). Early results showed that TZMbl cells do not endogenously express TLR2, consequently we established a stably transformed TZMbl cell line, TZMbl-2, that showed a substantial increase in TLR2 protein expression (Figure S1A in Supplementary Material). In addition, TLR1 protein was detected in TZMbl and TZMbl-2 cytosol fractions (Figure S1A in Supplementary Material). Importantly, TZMbl-2 exposed to TLR2 ligand, Pam_3_CSK_4_, produced significantly increased IL-8 levels in a dose-dependent manner compared to TZMbl (Figure S1B in Supplementary Material, *P* < 0.05, 0.001, 0.01, respectively). Taken together, these results indicated that TZMbl-2 cells expressed functional TLR2 and TLR1, and produced a pro-inflammatory response to synthetic bacterial TLR2/1 ligand, Pam_3_CSK_4_.

### Cellular TLR2 expression is associated with increased HIV-1 proviral DNA

Given that multiple viruses induce immune activation via TLR2 as a means to facilitate entry (36, 38, 39), as well as our recent paper which showed that soluble TLR2 (sTLR2) directly interacted with HIV-1 structural proteins (45), we explored whether cellular expression of TLR2 increased cell-free HIV-1 integration and viral-induced inflammation.

**Figure 1:**
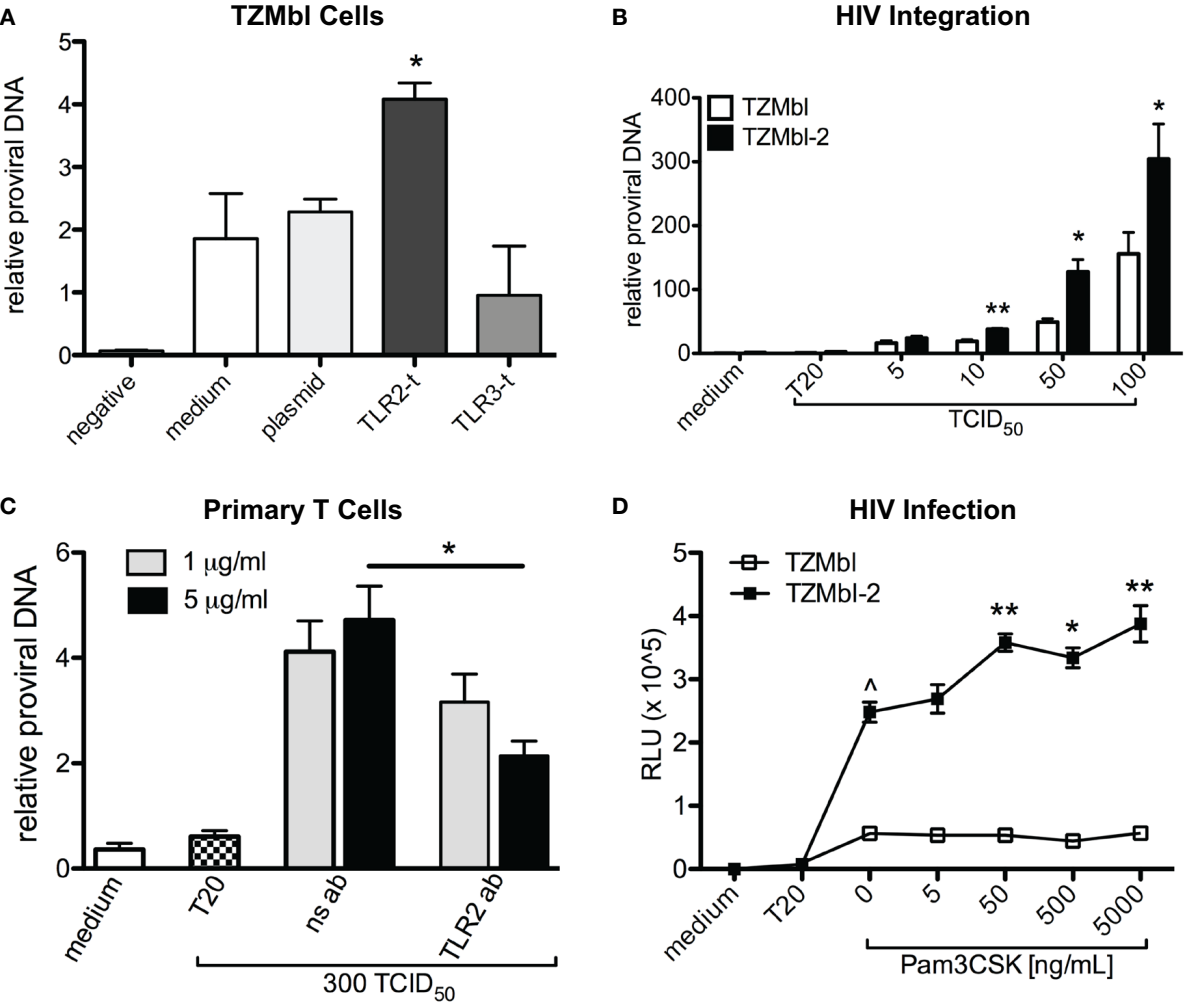
**Cellular TLR2 expression is associated with increased HIV-1 integration**. **(A)** TZMbl cells were transiently transfected with empty plasmid, TLR2, or TLR3 DNA and 24 h later were infected with 100 TCID_50_ of BAL (R5) virus. At 48 h post infection, DNA was isolated and analyzed for relative HIV-1 Pol proviral DNA to 18S rRNA gene. **(B)** HIV-1 Pol DNA was evaluated in TZMbl and TZMbl-2 exposed to various concentrations of virus at 8 h, and are the mean of triplicate samples ±SEM. **(C)** Primary CD4+ T cells were isolated from PBMCs and infected with 300 TCID_50_ of virus in the presence of non-specific antibody (ns ab) or anti-TLR2 antibodies (TLR2 ab) for 6 days, and then HIV-1 Pol DNA was evaluated. **(D)** TZMbl and TZMbl-2 cells were exposed to increased concentrations of Pam_3_CSK_4_ for 24 h prior to the addition of 100 TCID_50_ of virus. After 48 h, relative luciferase units (RLUs) were evaluated and showed significantly increased HIV infection in TZMbl-2 cells exposed to 5, 50, 500, and 5000 ng/mL of Pam_3_CSK_4_, prior to the addition of virus. A representative data set from three independent experiments is shown ±SEM. *P < 0.05, **P < 0.01, ***P < 0.001, ^^^*P* < 0.0001.

To first test our hypothesis, we transiently transfected TLR2, TLR3, or empty plasmid, into TZMbl cells and infected the cells with cell-free R5 HIV. Results revealed significantly increased HIV-1 proviral DNA compared to empty-plasmid control, whereas no significant increases in integration were identified in empty plasmid or TLR3-transfected TZMbl cells (Figure 1A; *P* = 0.0324).

Next, we infected TLR2 stably transformed cell line, TZMbl-2 and TZMbl with HIV-1 and evaluated viral DNA at 8 h post infection. These data indicated that TZMbl-2 had significantly increased proviral DNA compared to TZMbl cells at various infectious doses (Figure 1B; *P* = 0.0044, *P* = 0.017, *P* = 0.0132, respectively).

To directly investigate whether TLR2-specific activation preceding exposure to virus altered HIV infection, various concentrations of Pam_3_CSK_4_ were added to TZMbl-2 and TZMbl cells for 24 h prior to the addition of HIV-1. The results indicated significant increases in HIV infection in TZMbl-2 in a Pam_3_CSK_4_ dose-dependent manner (Figure 1C; *P* = 0.0041, 0.0137, 0.0051, respectively), while no significant increase was noted in TZMbl cells. Thus, TLR2 stimulation via Pam_3_CSK_4_ enhanced HIV infection.

Additionally, in primary human CD4+ T cells we tested whether neutralizing TLR2 using 1 or 5 μg/mL TLR2-specific antibodies could inhibit infection. We observed significant reduction in HIV DNA following treatment with 5 μg/mL of anti-TLR2 Ab compared to lymphocytes exposed to non-specific antibodies (Figure 1D; *P* = 0.0286).

Taken together these data indicated that cellular expression of TLR2 played an important role in significantly increased HIV-1 infection compared to cells that did not express TLR2, and TLR2 effects were blocked by anti-TLR2 Ab.

### HIV-1 p17 and gp41 induce pro-inflammatory cytokines through a TLR2-dependent mechanism

**Figure 2:**
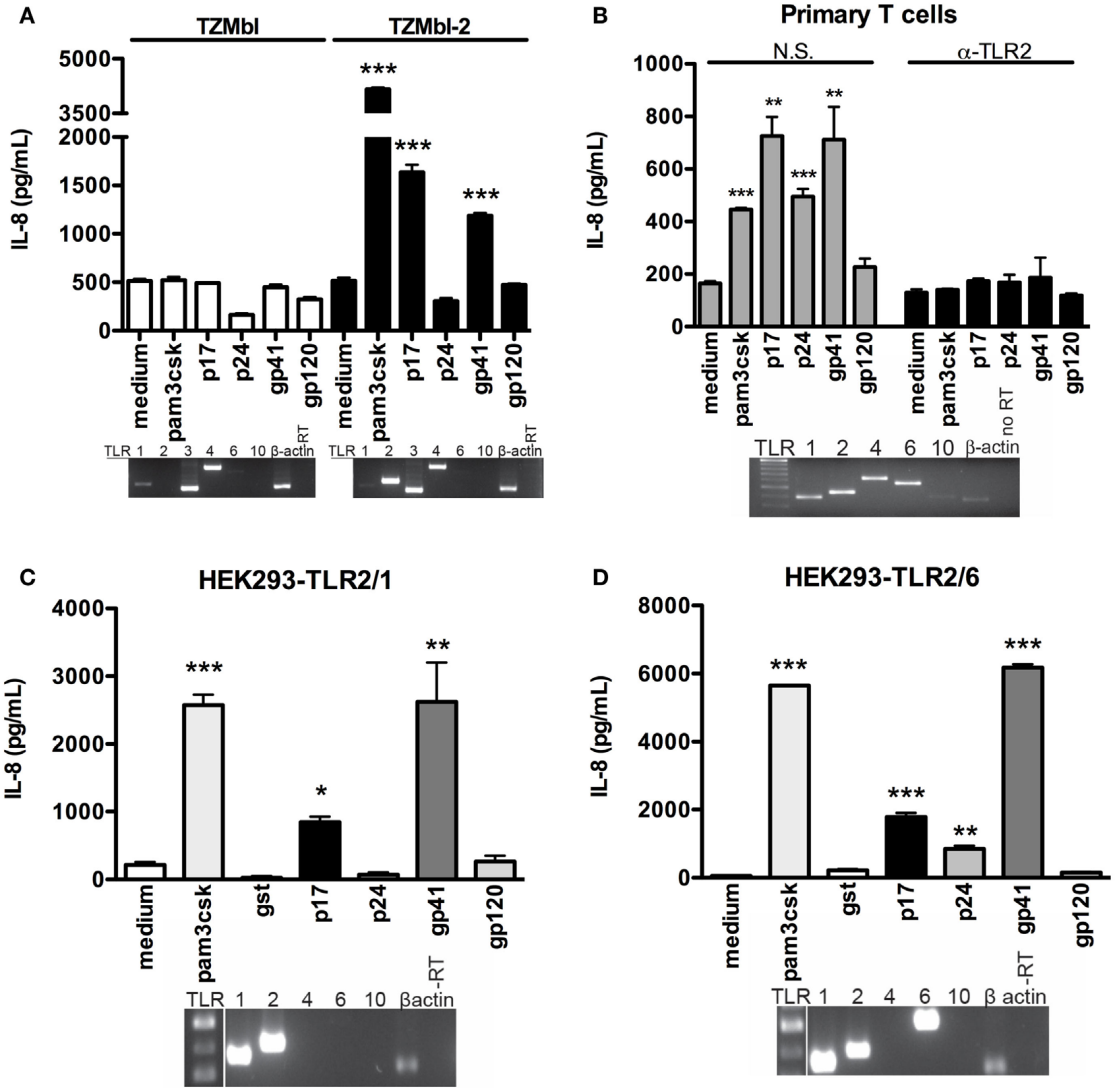
**HIV-1 protein-induced IL-8 stimulation was significantly increased in TLR2 transgene cell lines**. **(A)** TZMbl and TZMbl-2 cells, **(B)** primary CD3 + T cells were treated with non-specific (N.S.) or anti-TLR2 (α-TLR2) antibody for 1 h prior to treatment with 500 ng/mL TLR2 ligand (Pam_3_CSK_4_), 1 μg/mL HIV-1 proteins (p17, p24, gp41, gp120) or medium (control) and supernatants were collected after 4 h. **(C)** HEK293-TLR2/1 cells and **(D)** HEK293-TLR2/6 cells were treated overnight with 500 ng/mL TLR2 ligand (Pam_3_CSK_4_), 1 μg/mL HIV-1 proteins (p17, p24, gp41, and gp120), or medium (control) supernatants were evaluated for IL-8 production with ELISAs. Data are the mean of triplicate samples ±SEM. *P < 0.05, **P < 0.01, ***P < 0.001. Inserts: mRNA expression of TLR1, TLR2, TLR3, TLR4, TLR6, TLR10, and β-actin was detected in the cell lines with conventional RT-PCR as shown by electrophoresis agarose gels. A representative data set of at least three independent experiments is shown.

Numerous reports have highlighted the HIV-1 glycoproteins and matrix proteins’ role in inducing pro-inflammatory cytokines in both PBMC and specific epithelial cell subtypes (17–19). With this in mind, we set out to determine whether specific HIV-1 proteins induced immune activation as shown by pro-inflammatory cytokine production in cells expressing TLR2. Here TZMbl-2 and TZMbl cells were exposed to HIV-1 p17, p24, gp41, and gp120, positive control (Pam_3_CSK_4_) or negative controls (medium and protein tag glutathione transferase; GST). Results showed significantly increased IL-8 production in TZMbl-2 after exposure to p17, gp41, and Pam_3_CSK_4_ compared to medium, but not in TZMbl cells (Figure 2A, *P* = 0.0002, *P* = 0.0004, *P* < 0.0001, respectively). In contrast, p24 or gp120 protein exposure did not substantially increase IL-8 production in either TZMbl-2 or TZMbl cell lines (Figure 2A).

In Figure 2B insert, we showed that primary human CD3+ T cells endogenously express TLR1, 2, 4, 6, and 10. Importantly, in the absence of specific anti-TLR2 Ab, primary human T cells treated with p17, p24, gp41, gp120, and Pam3CSK4 demonstrated significantly increased IL-8 production compared to cells treated with gp120 or medium control (Figure 2B; *P* < 0.0001, *P* = 0.0015, *P* = 0.0004, *P* = 0.0099, respectively). In contrast, blocking of primary human T cells with anti-TLR2 Ab showed no significant changes of IL-8 production following treatment with HIV proteins or Pam_3_CSK_4_ (Figure 2B). To confirm our initial observation, we further investigated whether these specific HIV-1 proteins (p17, p24, gp41, and gp120) elicited pro-inflammatory responses in HEK293 cells expressing either TLR2/1 or TLR2/6. TLR2/1 cDNA expression levels were extremely high in HEK293-TLR2/1 cells (Figure 2C, insert), and they produced significantly elevated IL-8 levels after exposure to p17 and gp41 compared to medium, while p24 and gp120 did not induce a pro-inflammatory response (Figure 2C; *P* = 0.0023, *P* = 0.0143, respectively). HEK293-TLR2/6 cell line expressed cDNA for TLR2 and both heterodimers TLR1 and TLR6 (Figure 2D, insert). Exposure to various concentrations of recombinant viral proteins (p17, gp41, and p24) induced significantly increased levels of IL-8, while gp120 did not induce IL-8 compared to medium (Figure 2D; *P* = 0.0001, *P* = 0.0009, *P* < 0.0001, respectively). IL-8 was profoundly induced with gp41, likely due to higher amounts of TLR1/2/6 expression in HEK293-TLR2/6 cell line. As well, Pam_3_CSK_4_ (positive control) increased IL-8 production in both HEK293-TLR2/1 and HEK293-TLR2/6 cells (Figures 2C,D; *P* < 0.0001, *P* < 0.0001, respectively).

Taken together, these data indicated specific synergistic effects of the TLR2/1 heterodimer in sensing HIV proteins, p17 and gp41, and suggested that TLR2/6 heterodimer senses p24 leading to immune activation and production of pro-inflammatory cytokine. Further, the results confirm the importance of TLR2 in HIV protein-mediated activation of primary human T cells.

### siRNA knockdown of TLR2 and/or TLR1 inhibits HIV PAMP-induced cellular activation

**Figure 3:**
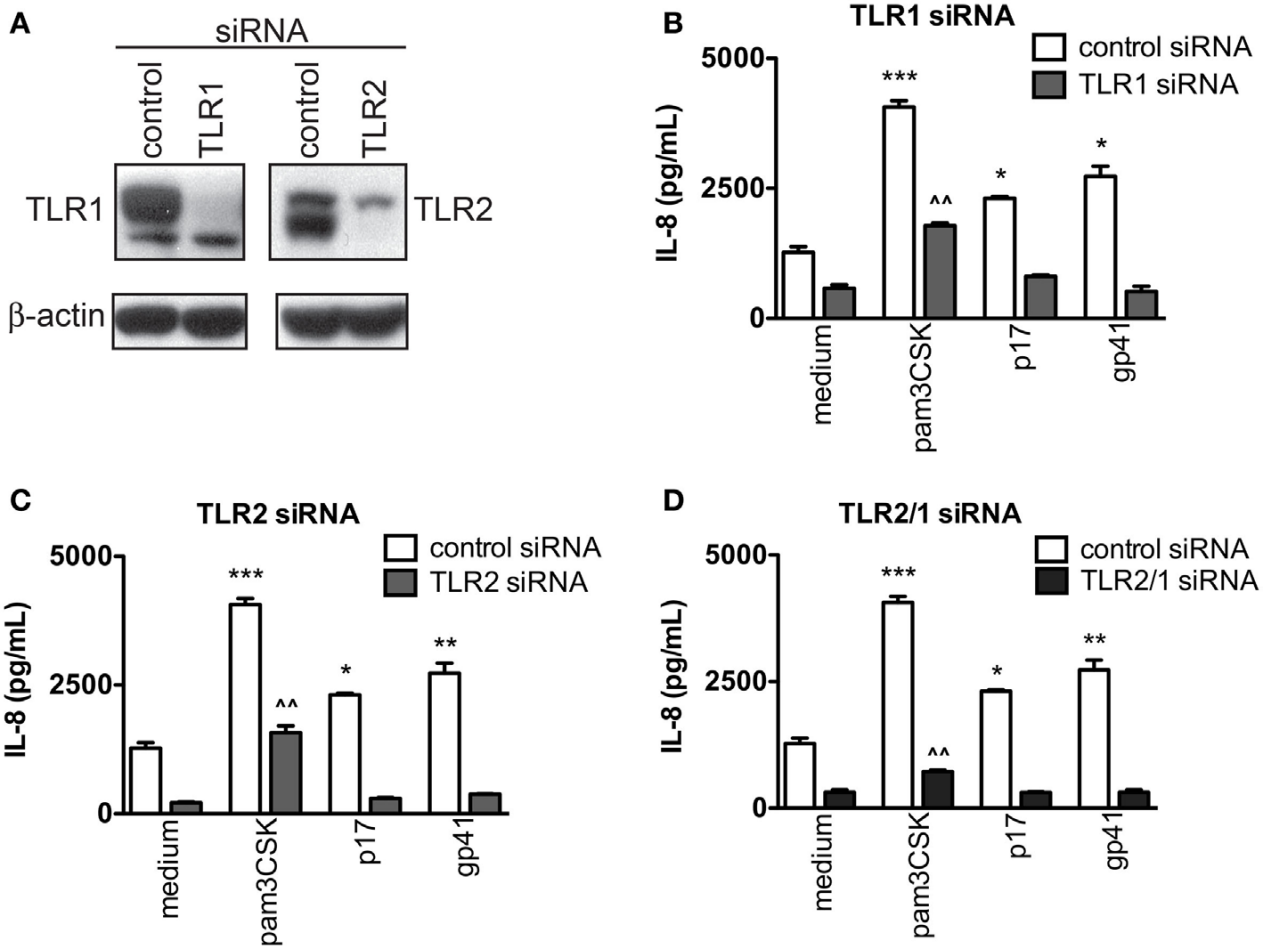
**HIV-1 p17- and gp41-induced IL-8 stimulation was significantly decreased in both TLR1 and TLR2 knocked down cells**. TZMb1-2 cells were transfected with 20 nM control siRNA, TLR1 siRNA, TLR2 siRNA or TLR2/1 siRNA for 2 days followed by medium containing 500 ng/mL Pam_3_CSK_4_ or 1 μg/mL HIV-1 proteins, p17 or gp41. **(A)** Western blot of TZMbl-2 cells 2 days after transfection with either TLR1 or TLR2 siRNA. Data in **(B–D)** represent IL-8 levels in supernatants, measured by ELISA after overnight culture, and are the mean of triplicate samples ±SEM. *P < 0.05, **P < 0.01, ***P < 0.001 compared to medium control; ^^^^*P* < 0.01 compared between medium and Pam_3_CSK_4_ in presence of TLR1, TLR2 or TLR2/1 siRNA. A representative data set of at least two independent experiments completed in triplicates is shown.

To understand the interaction by which HIV-1 proteins signal through TLR2 and/or its binding partner, TLR1, a series of siRNA knockdown assays were performed. Western blot analyses of total cell lysates showed substantially reduced endogenous TLR1 and TLR2 protein expression in TZMbl-2 cells treated with siRNA directed against TLR1 or TLR2 compared to non-specific control siRNA (Figure 3A). After siRNA knockdown, TZMbl-2 cells were exposed to viral proteins overnight and IL-8 levels were assessed. Results indicated that single siRNA knockdown of TLR1 or TLR2 ablated viral p17 and gp41-induced IL-8 production, as well as significantly reduced Pam_3_CSK_4_-induced IL-8 levels (Figure 3B; *P* = 0.0004, *P* = 0.0119, *P* = 0.0152; *Reduction*, *P* = 0.0051 and Figure 3C; *P* = 0.0004, *P* = 0.0119, *P* = 0.0074; *Reduction*, *P* = 0.0092). Little to no IL-8 response was elicited by Pam_3_CSK_4_, p17 or gp41 following treatment with a combination of TLR1/TLR2 siRNA (Figure 3D; *P* = 0.0004, *P* = 0.0119, *P* = 0.0074; *Reduction*, *P* = 0.0052). These results confirmed that both TLR1 and TLR2 are involved in recognizing these specific HIV-1 structural proteins.

Collectively, these data provided strong evidence that HIV-1 proteins, p17 and gp41, act as PAMPs and have a structural signature recognized by TLR2/1 heterodimer that activated innate immune responses in host cells.

### HIV-1 proteins bind TLR2 from TZMbl-2 cell lysates

**Figure 4:**
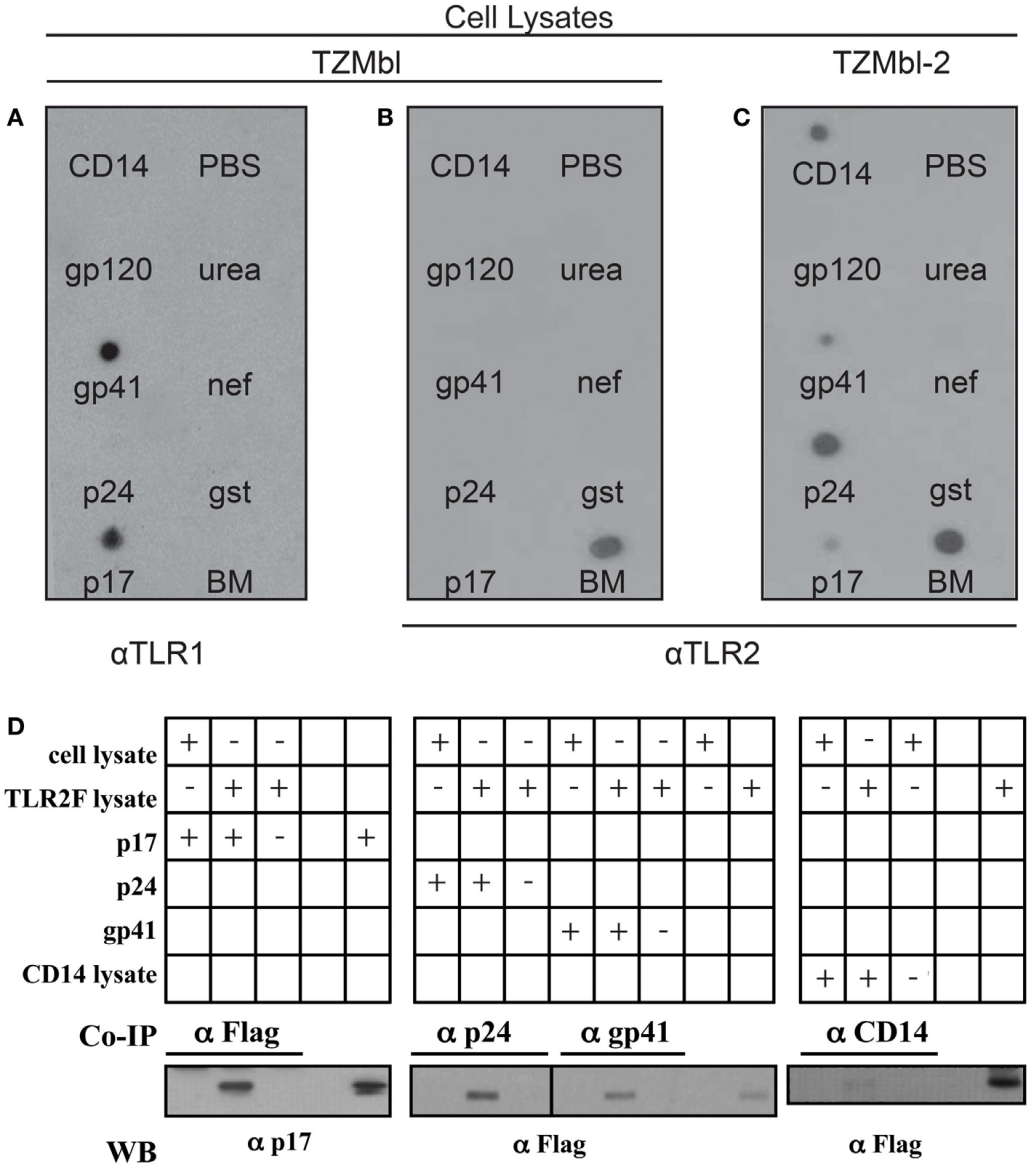
**HIV-1 proteins bind TLR2 from TZMbl-2 cell lysates**. **(A)** TZMbl cell cytoplasm, **(B)** TZMbl cell membrane fraction, and **(C)** TZMbl-2 cell membrane fraction were incubated with nitrocellulose membranes previously blotted with positive control (20 pmol sCD14, and 1 μL 1:20 diluted breast milk (BM), HIV-1 components (20 pmol; p17, p24, gp41, gp120, and nef), protein tag (20 pmol GST) and negative controls (1 μL PBS and 1 μL 1 M urea) and developed using α-TLR1 antibody in **(A)** and α-TLR2 antibody in **(B,C)**. **(D)** Immunoprecipitation of specific HIV proteins or CD14 (positive control) with TLR2 from cell lysates. TLR2/HIV protein complexes were co-IP with anti-flag (α-flag), anti-p24 (α-p24), anti-gp41 (α-gp41), or anti-CD14 (α-CD14) antibodies and harvested with protein G-beads. Co-IP interactions were identified between TLR2 and HIV-1 proteins or CD14 after separation by SDS-PAGE and staining using α-p17 or α-Flag antibodies, respectively. In all panels, aliquots of cell lysate containing TLR2 or recombinant proteins were conducted in parallel for identification of specific protein bands. A representative data set of at least three independent experiments in shown.

Two approaches were utilized to determine physical protein-to-protein interaction between TLR2 and specific HIV-1 proteins. Initially a dot blot detection method was used, as previously described (50). Recombinant HIV-1 proteins (p17, p24, gp41, gp120, and nef), positive controls CD14 and 1:20 diluted human breast milk (BM), which contains high levels of sTLR2 (41, 44), as well as negative carrier controls (urea solution, PBS, GST) were blotted onto nitrocellulose membrane. After blocking, the membranes were exposed to TZMbl or TZMbl-2 cell lysates and viral protein–TLR interactions were identified using TLR2 or TLR1-specific antibodies. Results indicated that HIV-1 p17, p24, gp41, and pure CD14 directly interacted with membrane bound TLR2, whereas no interaction was detected between TLR2 and gp120, nef, or ssRNA40 (Figure 4A). TZMbl lysate incubation did not reveal any interactions with viral proteins, or CD14 control, however the membrane control was positive, thus demonstrating that there was no non-specific antibody binding (Figure 4B). Furthermore, TZMbl lysates probed with anti-TLR1 antibodies indicated strong interactions between TLR1 and viral proteins p17 and gp41, but not with p24 or other viral components (Figure 4C).

Co-immunoprecipitation (co-IP) assays validated our findings and demonstrated that TLR2 interacted with p17, p24, gp41, and CD14 (Figure 4D). Taken together, these data demonstrate, for the first time, direct protein-to-protein interactions between TLRs and HIV-1 structural proteins in which TLR2 preferentially bound p17, p24, and gp41 while TLR1 only interacted with p17 and gp41.

### HIV-1 proteins activate NFκB signaling in TZMbl-2 and primary human T cells

**Figure 5:**
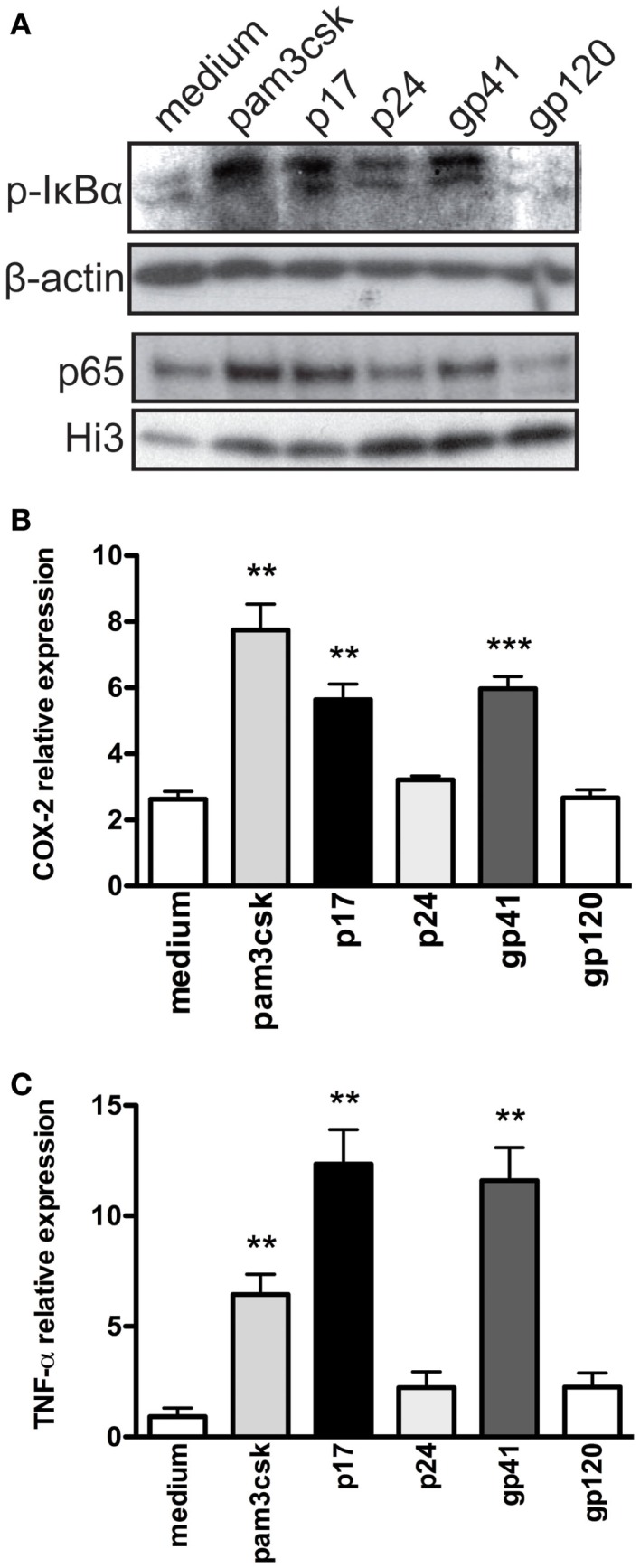
**HIV-1 proteins activate phosphorylation of IκBα and trigger pro-inflammatory cytokine production in TZMbl-2 cells**. **(A)** Cell lysates from TZMbl-2 were evaluated for phosphorylated IκBα and p65 nuclear translocation using western blot analyses after treatment with 500 ng/mL positive control (Pam_3_CSK_4_), 1 μg/mL HIV-1 proteins (p17, p24, gp41, and gp120), and negative control (medium) for 2 h, respectively. Evaluation of **(B)** COX-2 and **(C)** TNF-α expression in TZMbl-2 treated as in **(A)** except for 4 h. For data, not less than three independent experiments were analyzed.

Recognition of PAMPs by TLRs typically induce pro-inflammatory responses via the phosphorylation of IκBα, the inhibitory subunit of NFκB, and subsequent translocation of NFκB subunits p50/p65 into the nucleus (32). Given the central role of NFκB to activation of innate immune responses, we sought to qualitatively assess the impact of HIV-1 protein exposure on the phosphorylation of IκBα in TZMbl-2 cells and primary human T cells. Following exposure to HIV-1 proteins (p17, p24, gp41, and gp120), positive control (Pam_3_CSK_4_), and negative control (medium), western blot analysis of TZMbl-2 cell lysates showed a substantial increase in phosphorylated IκBα in cells exposed to p17, gp41, and Pam_3_CSK_4_, compared to medium and gp120 (Figure 5A). Furthermore, assessment of NFκB subunit p65 nuclear translocation in TZMbl-2 cells revealed substantially increased p65 in nuclear fractions of TZMbl-2 cells that were exposed to p17, gp41, and Pam_3_CSK_4_, but not in cells exposed to medium or p24 (Figure 5A).

Given that HIV-1 infection induces COX-2 in multiple cell types (52, 53), we examined COX-2 mRNA in TZMbl-2 cells exposed to HIV-1 proteins (p17, p24, gp1, and gp120), positive control (Pam_3_CSK_4_), and negative control (medium). Our results showed significantly increased COX-2 cDNA expression levels in TZMbl-2 cells exposed to p17, gp41, and Pam_3_CSK_4_ compared to medium (Figure 5B; *P* = 0.0033; *P* = 0.0022; *P* = 0.0002, respectively). As well, TNF-α is strongly implicated in HIV-1 pathogenesis (54); therefore, we examined cDNA expression using qRT-PCR and showed significantly increased TNF-α expression in TZMbl-2 cells exposed to p17 and gp41, and Pam_3_CSK_4_ compared to medium (Figure 5C; *P* = 0.0058, *P* = 0.0064, *P* = 0.0051, respectively).

**Figure 6:**
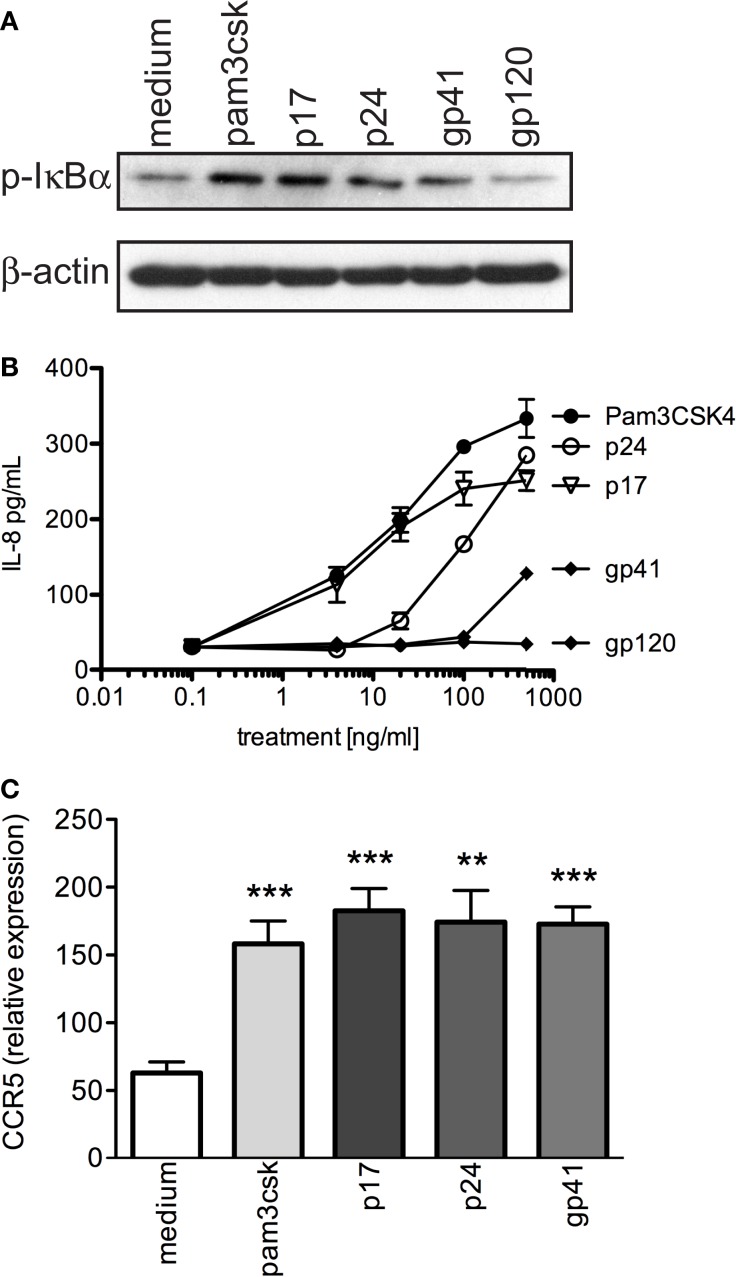
**HIV-1 structural proteins activate primary T cells and significantly increase CCR5 expression**. **(A)** Western blot of p-IκBα expression in primary human T cells after exposure to 500 ng/mL positive control (Pam_3_CSK_4_), 1 μg/mL HIV-1 proteins (p17, p24, gp41, and gp120), and negative control (medium) for 2 h. **(B)** IL-8 production was evaluated in the supernatants of primary human T cells that were exposed to increasing concentrations of TLR2 ligand, Pam_3_CSK_4_, as well as HIV-1 proteins (p17, p24, gp41, and gp120) for 10 h. **(C)** Evaluation of CCR5 cDNA expression in CD3+ primary human T cells. A representative data set of at least three independent experiments is shown completed in triplicate samples ±SEM. *P < 0.05, **P < 0.01, ***P < 0.001.

Next, we examined primary human T cells exposed to specific HIV-1 structural proteins, p17, p24, gp41, or positive control, Pam_3_CSK_4_ and observed significantly increased phosphorylation of IκBα (p-IκBα) (Figure 6A). We then evaluated the level of IL-8, as an indicator of primary T cell activation, and observed that p17 elicited significantly increased production of the chemotactic cytokine at equivalent concentrations of TLR2 PAMP, Pam_3_CSK_4._ In contrast, p24 and gp41 activated primary T cells to produce substantial amounts of IL-8, but required 5- and 25-fold, respectively, greater concentrations compared to p17 and Pam_3_CSK_4_ (Figure 6B). Thus, activation of primary human T cells by gp41 required a significantly greater dose compared to p17 and p24. Lastly, gp120 produced basal IL-8 levels at all doses tested.

It has previously been shown that the heightened state of cellular activation of HIV-1 infected individuals is associated with increased CCR5 expression in CD4+ T cells, which may help propagate R5 viruses (55). In addition, Heggelund et al. (56) previously demonstrated that direct TLR2 stimulation significantly increased CCR5 protein expression in primary mononuclear cells. Thus, we next determined whether primary T cells exposed to viral proteins (p17, p24, gp41, and gp120) or Pam_3_CSK_4_ increased co-receptor expression, and observed that CCR5 cDNA expression was significantly elevated after 4 h of exposure to p17, p24, gp41, as well as Pam_3_CSK_4_ compared to medium (Figure 6C; *P* = 0.0014, 0.0256, 0.0027, respectively).

Together, these data indicate that specific HIV-1 structural proteins (p17, p24, and gp41) induce significant cellular activation and increased HIV-1 co-receptor expression in primary human T cells.

### p24 blocks p17 and gp41-induced production of pro-inflammatory cytokines

Data shown above (Figures 4A,B) indicated a strong protein-to-protein interaction between p24 and TLR2, yet did not induce a pro-inflammatory response in TZMbl-2 cells that lack expression of TLR6. Therefore, we sought to determine whether p24 was interacting with TLR2 in a manner that impacted TLR2-dependent pro-inflammatory activation induced by p17 and gp41.

**Figure 7:**
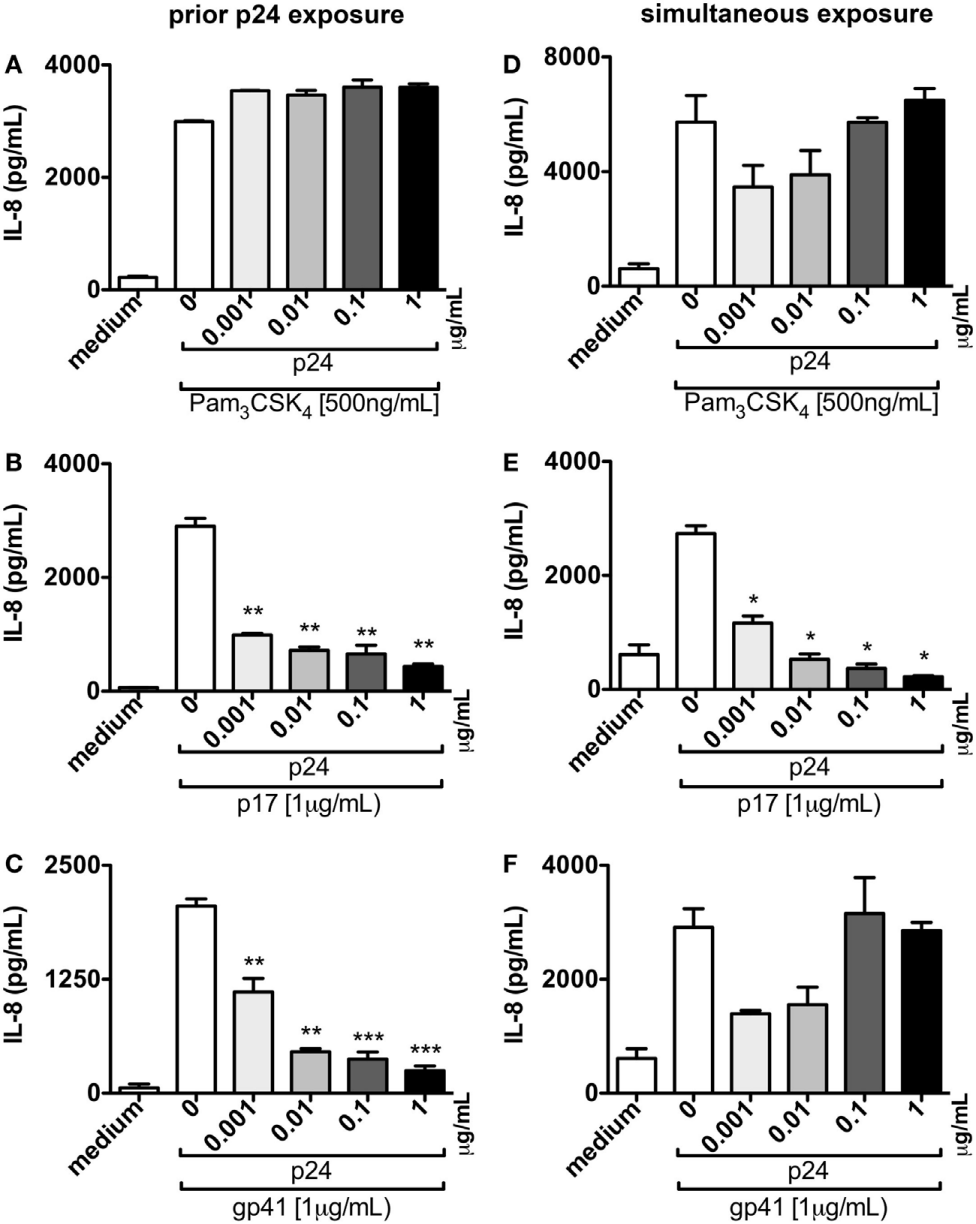
**p24 blocks p17 and gp41-induced production of pro-inflammatory cytokines**. **(A–C)** TZMbl-2 cells were pre-treated with various doses ranging from 0 to 1 μg/mL of p24 before 500 ng/mL TLR2 ligand (Pam_3_CSK_4_), or 1 μg/mL HIV-1 structural proteins, p17 or gp41 were added. **(D–F)** TZMbl-2 cells were simultaneously exposed to various doses of p24 and Pam_3_CSK_4_ or HIV-1 structural proteins at the doses noted above. Data in **(A–F)** represent IL-8 concentrations in supernatants that were measured in ELISAs after overnight culture, and are the mean of triplicate samples ±SEM. *P < 0.05, **P < 0.01, ***P < 0.001. A representative data set from three independent experiments is shown.

TZMbl-2 cells were incubated with increasing concentrations of p24 for 1 h prior to the addition of cellular activators, Pam_3_CSK_4_, p17, or gp41, and resulted in a significant dose-dependent attenuation in IL-8 production following exposure to p17 and gp41 (Figures 7B,C; *P* = 0.0054, *P* = 0.0047, *P* = 0.0016, *P* = 0.0034, *P* = 0.011, *P* = 0.0028, *P* = 0.0006, *P* = 0.0003, respectively), yet had little to no effect on the inhibition of Pam_3_CSK_4_-induced production of IL-8 (Figure 7A).

Next, TZMbl-2 cells simultaneously exposed to p24 and Pam_3_CSK_4_, p17, or gp41 indicated that p24 did not inhibit Pam_3_CSK_4_ or gp41 cellular activation (Figures 7D,F). However, p24 did significantly block p17-induced production of IL-8 in a dose-dependent manner (Figure 7E, *P* = 0.0135, *P* = 0.0473, *P* = 0.0421, *P* = 0.0345, respectively).

Collectively, these data suggest that p24 can bind to TLR2 and block activation by p17 and gp41 HIV structural proteins, but not Pam_3_CSK_4_. Thus providing a novel mechanism capable of manipulating innate immune signaling through a TLR2-dependent mechanism.

## Discussion

**Figure 8:**
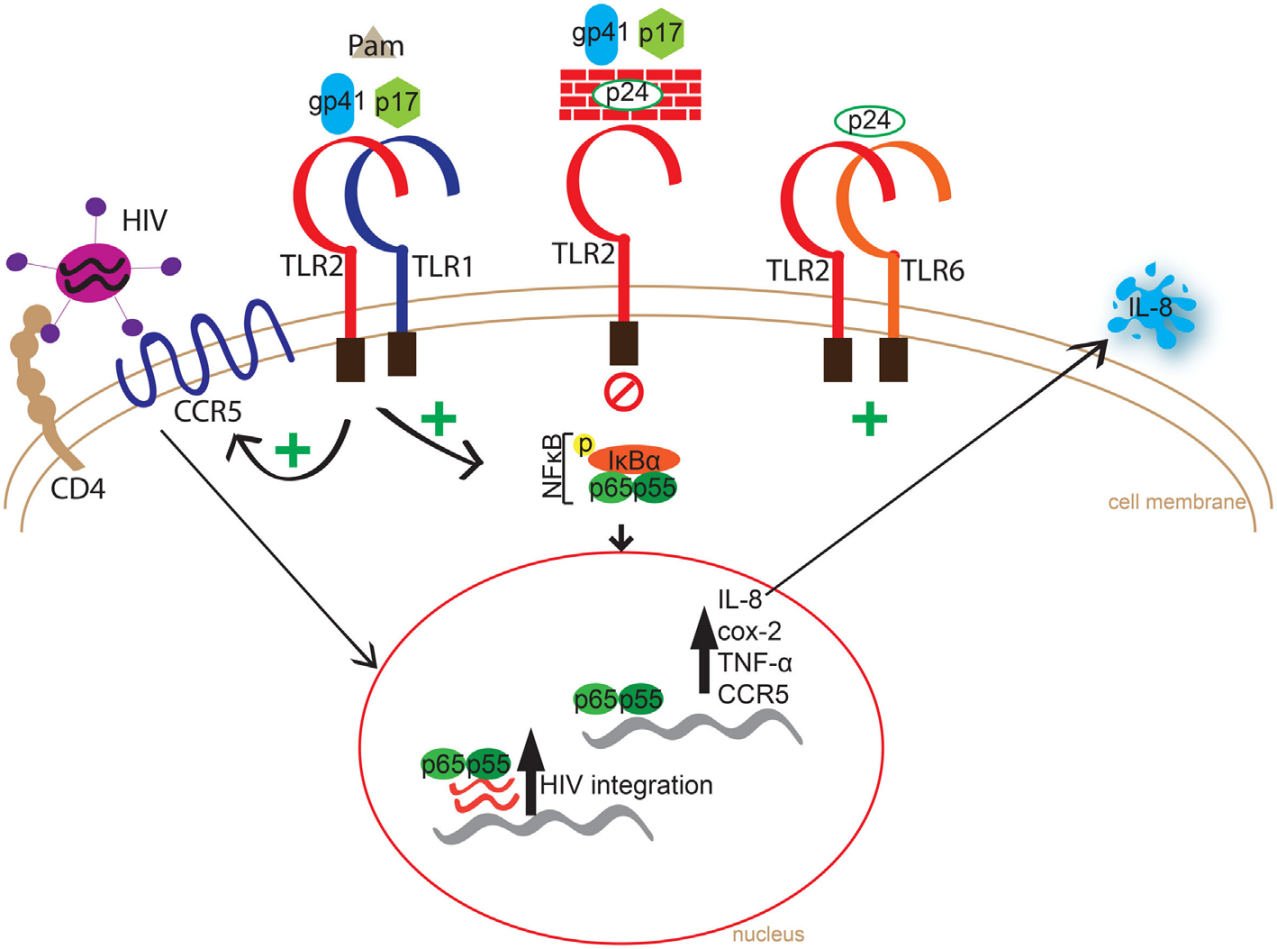
**Schematic representation of HIV PAMPs interaction and immune activation via TLR2 homo or heterodimers**. Innate immune recognition of HIV-1 proteins, p17 and gp41, through TLR2/1 results in phosphorylation of IκBα leading to amplified transcription of IL-8, COX-2, TNF-α, and CCR5, and increased IL-8 levels. In the absence of TLR6 expression, p24 blocked p17 and gp41-induced production of pro-inflammatory cytokine, yet induced the production of IL-8 in cells expressing the TLR2/6 heterodimer. Cellular TLR2 expression significantly increased HIV integration, possibly due to an increase in CCR5 co-receptor expression.

Chronic immune activation is a central driver of HIV-1 infection and progression to AIDS (1, 2, 4–6), and can be partially attributed to the rapid and massive depletion of gastrointestinal CCR5+CD4+ T cells following infection (57, 58), which compromises the integrity of the mucosal barrier and facilitates translocation of bacteria from the gut (1, 2, 59). Indeed, increased lipopolysaccharide (LPS) levels in sera, a result of microbial translocation through the gut-associated lymphoid tissue (GALT), has been documented to strongly correlate with immune activation in chronically HIV-1-infected individuals (59). However, we previously demonstrated that following cART and subsequent decrease in viral load, TLR expression in previously untreated HIV-infected individuals normalized, thus suggesting that HIV itself may be involved in increased activation (12). Additionally, we recently showed that soluble TLR2 (sTLR2), which is found in human milk, other mucosal fluids, and systemically, inhibited HIV infection *in vitro* (44), through a direct interaction with HIV-1 structural proteins, and inhibited virally induced NFκB activation and inflammation (45). These findings led us to ask a rather simple but important question, namely, do HIV-1 structural proteins act as PAMPs for TLR2 and its heterodimers? Here, we tested a number of HIV-1 structural proteins as PAMPS, and demonstrated that specific HIV proteins can engage and activate membrane bound TLR2 heterodimers, while others block TLR2 activation. Moreover, we demonstrated that cellular TLR2 expression significantly increased HIV-1 infection/integration *in vitro*. Collectively, we provide an illustrated summary of our results highlighting HIV PAMPs interactions with TLR2 heterodimer signaling, leading to increased immune activation, CCR5 expression, and HIV integration (Figure 8).

The role of TLRs in HIV pathogenesis has not been fully elucidated; however, multiple manuscripts have described the importance of HIV-1 specific cellular activation through TLRs that facilitates viral replication in pDC and T cells (25–27, 60, 61). Specifically, activation of TLR2 has been shown to increase viral replication and the production of pro-inflammatory cytokines in naïve and memory T cells (40, 61, 62). To investigate the role of TLR2 directly sensing HIV-1 structural proteins and contributing to increased infection and pro-inflammatory cytokine production, we tested primary human T cells and a TLR2 stably transformed TZMbl cell line (TZMbl-2). Our data demonstrate that expression of TLR2 significantly increases HIV integration and production of pro-inflammatory cytokines. These data support earlier investigations that described increased TLR2 expression on primary cells from HIV-infected patients that when activated through this specific TLR led to increased viral replication and TNF-α production (56), which suggests an important role for TLR2 in innate immune activation. Indeed, cellular exposure to specific HIV-1 structural proteins, namely p17, p24, and gp41 significantly increased NFκB-induced cellular activation and inflammation, which could be knocked down following pretreatment with anti-TLR2 antibodies or TLR2/1 specific siRNA. Interestingly, not all the HIV-1 structural proteins tested triggered cellular activation through the same heterodimer pair. We demonstrated that cells expressing TLR2/1 responded to p17, gp41, and Pam_3_CSK_4_, but not p24 or gp120, while TLR2/6 heterodimer led to production of pro-inflammatory cytokines in the presence of p24. Previous publications have provided evidence that multiple HIV-1 proteins play key roles in altering cellular activity. Indeed, p17 has previously been shown to induce the production of pro-inflammatory cytokines *in vitro* and *in vivo* (17, 63), and act as an adjuvant in vaccine strategies tested in animal models (64). Furthermore, CXCR1 has been identified as a p17 receptor that triggered adhesion and chemotactic-like migration in monocytes (65). Given that CXCR1 is not expressed on many epithelial cell types, including HeLa (66), the data provided here revealed TLR2 as a novel innate HIV structural protein PRR. Importantly, we previously published on the fact that our protein preparations showed little to no endotoxin contamination (45), and that viral proteins were sensitive to proteinase K digestion, thus further supporting that TLR2 stimulation by HIV-1 PAMPs was not due to LPS contamination. Moreover, siRNA knockdown of TLR2 and/or TLR1 but not TLR4 ablated the production of pro-inflammatory cytokines after HIV-1 PAMP exposure, and HIV-1 PAMPs did not induce production of IL-8 in TZMbl cells which endogenously express TLR4 but not TLR2. Furthermore, TLR2 antibodies abrogated IL-8 production in primary T cells, and HEK293 cell lines do not express TLR4, yet responded to specific HIV proteins in a TLR2-dependent manner. These results indicate a novel mechanism in which HIV-1 structural proteins induce immune activation through TLR2 heterodimers that was not due to endotoxin contamination.

Recognition of viral proteins by TLR2 heterodimers is not unique to HIV-1. A number of reports highlight the importance of TLR2 expression in sensing multiple viral proteins that led to increased cellular activation and facilitated viral entry (36–39). However, previous publications demonstrated a TLR2-mediated enhancement of HIV integration in resting T cells (67). Interestingly, our evaluation of PRR expression in a population of highly exposed seronegative (HESN) female commercial sex workers (CSWs) showed significantly decreased expression of TLR2 in cervical epithelial and cervical mononuclear cells compared to HIV-uninfected and HIV-infected CSWs from the same cohort (48). Here, we showed that expression of TLR2 significantly increased HIV integration compared to cells that did not express this particular PRR *in vitro*. Interestingly, in a recent study evaluating TLR polymorphisms associated with HIV outcomes in Sub-Saharan Africans, it was shown that a single nucleotide polymorphism (SNP) in TLR2 was associated with HIV-1 set-point (68). Together indicating that TLR2 may play an important role in viral infection, particularly HIV, and needs to be investigated further.

For us, the question remained, what was the mechanism by which HIV-1 induces cellular activation leading to increased viral integration through TLR2? Using two approaches, a dot blot detection method and co-immunoprecipitation, we examined the direct protein-to-protein interactions between TLRs and HIV-1 structural proteins and showed that they physically associated. Specifically, structural proteins, p17, p24, and gp41, bound directly to TLR2, while only p17 and gp41 interacted with TLR1, indicating that one of the functional sensors for HIV-1 is a TLR2/1 heterdimeric complex. Indeed, this would help explain why primary cells of HIV-1 infected individuals have significantly increased TLR2 expression compared to uninfected individuals (12, 56).

Following the physical interaction between p17, p24, or gp41 and TLR2, there was a substantial increase in phosphorylated IκBα in primary and transformed cell lines compared to medium, which led to nuclear translocation of NFκB subunit p65. This is an important step not only in the TLR2 signaling pathway but is also required for induction of HIV-1 gene expression via viral LTR binding (69). Moreover, primary human T cells showed significantly increased COX-2 and TNF-α expression in a TLR2-dependent manner, and importantly a TLR2-dependant increase in HIV co-receptor, CCR5 expression in primary T cells. These observations are in line with previous accounts of a TLR2-dependent increase of CCR5 expression in permissive cells (56, 67). These data may provide a key mechanism for increased susceptibility to HIV infection, and help explain why expression of TLR2 significantly increased HIV integration in permissive cells while providing important information about the innate immune mechanism by which HIV-1 proteins can promote infection through increased CCR5 expression on target cells.

Our data showing that in the absence of TLR6, p24 blocked p17- and gp41-induced pro-inflammatory cytokine production in a dose-dependent manner was an unexpected finding, but are particularly intriguing since they provide evidence for a mechanism by which HIV-1 can manipulate the innate immune balance between viral protein-induced immune activation and inhibition in a TLR2-dependent manner. p24 was unable to block Pam_3_CSK_4_-induced immune activation, which is also intriguing since it is well documented that Pam_3_CSK_4_ binds in the crevice of the m-shaped heterodimer produced through the interaction of TLR2 and TLR1 (70, 71). Therefore, we speculate that HIV proteins bind to alternate conformations or in different regions of this heterodimer than previously described for Pam_3_CSK_4_ (41). Another unexpected outcome was that gp120 had little to no effect on cytokine production or IκBα phosphorylation, which was unexpected given recent publications showing the induction of pro-inflammatory cytokines in genital epithelial cells (72). This suggests that cellular responses to innate activation by HIV may be contextual and thus requires further investigation.

Previous publications have shown that gp41 activated NFκB in exposed lymphocytes and gp120 altered inflammatory responses *in vitro* (73, 74). Importantly, it was shown by Popovic et al. (15) that HIV structural proteins and glycoproteins were present and persisted in germinal centers of lymph nodes in abundant amounts both before and during cART (15). Furthermore, numerous studies have shown the presence of extracellular or virus-free HIV-1 proteins present in body fluids (75, 76), e.g., extracellular Vpr was detected in serum of HIV-infected individuals at levels of 5–10 ng/mL (77). Taken together with data indicating HIV-1 infected cells continually shed large amounts of viral proteins as well as defective viral particles (78), it seems critical for future experiments to determine the role this viral milieu provides in driving innate activation and HIV-related pathogenesis in HIV-infected individuals.

In conclusion, the present investigation extends our current understanding of innate sensing of HIV-1 and for the first time reveals novel HIV-1 PAMPs, including p17, p24, and gp41, that can manipulate innate sensing and cellular activation. Further, our finding that TLR2 expression significantly increased HIV integration via increased CCR5 expression provides a mechanism by which HIV-1 can regulate host infection and persistence. Perhaps by manipulating TLR2-related innate activation, one can significantly decrease the reservoir of latently HIV-infected cells. Additionally, the data shown here indicated that TZMbl-2 cells are more highly activated after exposure to HIV-1 structural proteins and have significantly increased HIV integration rates compared to TZMbl cells. Therefore, TZMbl-2 cells might be more suitable compared to TZMbl cells for assessing HIV-1 infectivity, integration and, measurement of effectiveness of neutralizing antibodies since TZMbl cells may underestimate the level of viral infection and integration. Lastly, identification of these novel HIV PAMPs has potential implications for the development of HIV vaccines. Indeed, HIV proteins that serve as innate PAMPs have auto-adjuvant activity and thus may prove to be a more effective antigen delivery systems to promote immunogenicity of HIV vaccines. Together, these results have important implications for our fundamental understanding of innate immune activation by HIV-1 and may provide insight into the design of novel vaccine strategies, as well as targeting latently infected cells to bring us one step closer to HIV cure.

## Conflict of Interest Statement

The authors declare that the research was conducted in the absence of any commercial or financial relationships that could be construed as a potential conflict of interest.

## Supplementary Material

The Supplementary Material for this article can be found online at http://journal.frontiersin.org/article/10.3389/fimmu.2015.00426

## Funding

This work was supported by a Large Team grant from the Canadian Institutes of Health Research (CIHR) to KR (PI) as part of the Canadian HIV Vaccine Initiative (CHVI). The funders had no role in study design, data collection and analysis, decision to publish, or preparation of the manuscript.
