# Supplementary material for: HIV-1 Structural Proteins Serve as PAMPs for TLR2 Heterodimers Significantly Increasing Infection and Innate Immune Activation
Source: Front Immunol. 2015 Aug 19;6:426. doi: 10.3389/fimmu.2015.00426 (PMC4541371; doi:10.3389/fimmu.2015.00426)
Supplement: Supplementary file 1 [file Image_1.PDF]

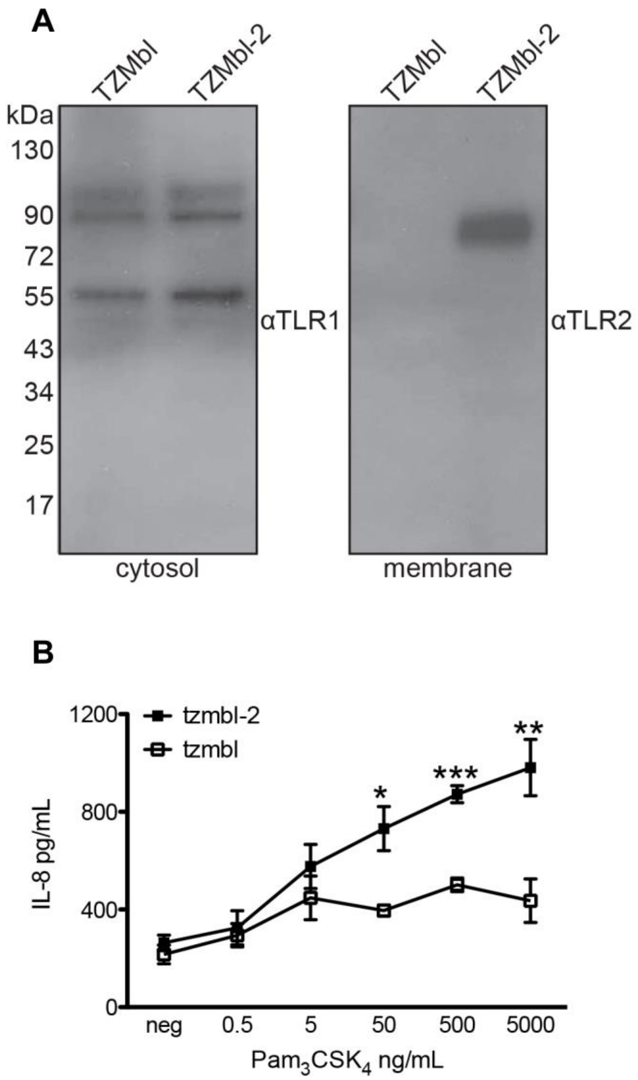

Supplementary Figure 1. Establishment of a functional TLR2 stably transfected cell line, TZMbl-2. (A) TLR1 and TLR2 cellular protein expression in TZMbl and TZMbl-2 cell fractions were evaluated by western blot analyses using anti-TLR1 antibodies (α TLR1) and anti-TLR2 antibodies (α TLR2). (B) TZMbl and TZMbl-2 cell supernatants were assessed for IL-8 production after exposure to various concentrations of Pam_3CSK_4. *$P<0.05$, **$P<0.01$, ***$P<0.001$. Errors bars, SEM. A representative data set from triplicate experiments is shown.
