# Supplementary material for: HIV-1 Structural Proteins Serve as PAMPs for TLR2 Heterodimers Significantly Increasing Infection and Innate Immune Activation
Source: Front Immunol. 2015 Aug 19;6:426. doi: 10.3389/fimmu.2015.00426 (PMC4541371; doi:10.3389/fimmu.2015.00426)
Supplement: Supplementary file 2 [file Image_2.PDF]

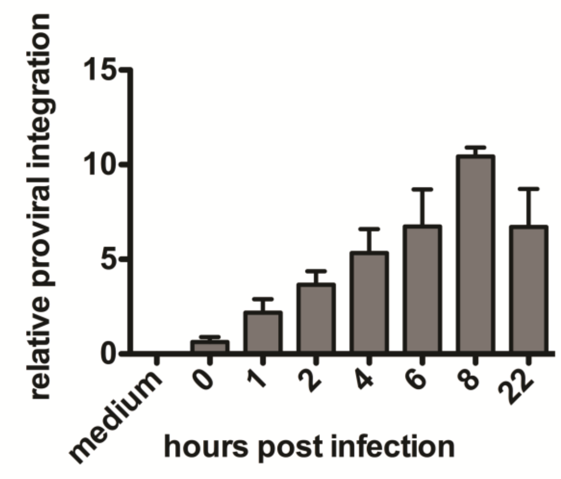

Supplementary Figure 2. Optimization of HIV-1 integration in TZMbl cell line. TZMbl cells were exposed to 100 $TCID_{50}$ of BAL (R5) virus and DNA was isolated at various time points (0, 2, 4, 6, 8, 22 hours) post infection and HIV-1 integration determined by qRT-PCR. Data are the mean of triplicate samples ± SEM. No less than two independent experiments were analyzed.
